# Evaluation of Mannosidase and Trypsin Enzymes Effects on Biofilm Production of *Pseudomonas aeruginosa* Isolated from Burn Wound Infections

**DOI:** 10.1371/journal.pone.0164622

**Published:** 2016-10-13

**Authors:** Maryam Banar, Mohammad Emaneini, Mhboubeh Satarzadeh, Nafiseh Abdellahi, Reza Beigverdi, Willem B. van Leeuwen, Fereshteh Jabalameli

**Affiliations:** 1 Department of Microbiology, School of Medicine, Tehran University of Medical Sciences, Tehran, Iran; 2 Laboratory of Microbiology, Shahid Motahari Burns Hospital, Tehran, Iran; 3 Department of Medical Microbiology & Infectious Diseases. Erasmus Medical Center, University of Applied Sciences, Leiden, Netherlands; Laurentian, CANADA

## Abstract

Biofilm is an important virulence factor in *Pseudomonas aeruginosa* and has a substantial role in antibiotic resistance and chronic burn wound infections. New therapeutic agents against *P*. *aeruginosa*, degrading biofilms in burn wounds and improving the efficacy of current antimicrobial agents, are required. In this study, the effects of α-mannosidase, β-mannosidase and trypsin enzymes on the degradation of *P*. *aeruginosa* biofilms and on the reduction of ceftazidime minimum biofilm eliminating concentrations (MBEC) were evaluated. All tested enzymes, destroyed the biofilms and reduced the ceftazidime MBECs. However, only trypsin had no cytotoxic effect on A-431 human epidermoid carcinoma cell lines. In conclusion, since trypsin had better features than mannosidase enzymes, it can be a promising agent in combatting *P*. *aeruginosa* burn wound infections.

## Introduction

Burn wound infections are one of the most important complications that occur after burn injuries and may be associated with serious clinical complications and increased morbidity and mortality [1, 2]. *Pseudomonas aeruginosa* is one of the most important pathogens involved in burn infections [1]. The emergence of multidrug-resistant *P*. *aeruginosa* infections is the major concern with managing *P*. *aeruginosa* burn infections as it is very difficult to treat [3]. *P*. *aeruginosa* alters the expression of its virulence factors in wound infections [2], including the production of biofilm in burn wounds [4]. Such hospital-acquired infections delayed healing for 2 to 4 weeks [5]. The biofilm mediates bacterial stability and protects them from surrounding environment, such as the immune system and increases the antibiotic resistance [6].

The biofilm matrix in *P*. *aeruginosa* is composed of three distinct exopolysaccharides: alginate, Psl and Pel. Alginate is a polymer consisting of β-D-mannuronic acid and α-L-guluronic acid and has a substantial role in structural stability and protection of biofilm. Psl is a polysaccharide composed of a repeating pentasaccharide, consisting of D-mannose, D-glucose and L-rhamnose. Psl is important in the initiation of biofilm formation and protection of biofilm structure. Pel is the third polysaccharide which is present in *P*. *aeruginosa* biofilm and is glucose-rich [7]. Additionally, a lot of surface proteins are involved in *P*. *aeruginosa* biofilm formation [8].

Due to the increasing *P*. *aeruginosa* antibiotic resistance and given the importance of biofilm in increasing the antimicrobial resistance, researchers are exploring novel therapeutic strategies targeting biofilms. This may contribute to improve the treatment of biofilm-related infections [9]. Some of the anti-biofilm methods that have been studied in recent years include: small molecule based inhibitors, phytochemicals, bacteriophage therapy, photodynamic therapy, antimicrobial peptides, monoclonal antibodies, nanoparticles and biofilm degrading enzymes [10–13].

The α-mannosidase enzyme is an acid hydrolase which is located in plant vacuoles and is thought to be involved with the turnover of N-linked glycoproteins and has been purified from Canavalia ensiformis (Jack bean) [14]. The β-mannosidase enzyme was purified from helix pomatia and hydrolyzes the terminal mannose residues, which are β-1→4 linked to oligosaccharides or glycopeptides [15]. Based on the structure of Psl polysaccharide and due to the performance features of mannosidase enzymes, it was assumed that these enzymes may destroy Psl polysaccharide.

Trypsin is a pancreatic serine endoprotease that cleaves proteins or peptides on the carboxyl side of arginine (R) or lysine (K) residues [16]. It was supposed that trypsin enzyme may destroy protein contents of the biofilm matrix in *P*.*aeruginosa*.

In the current study, we investigate the effects of mannosidase and trypsin enzymes on the degradation of biofilms of *P*. *aeruginosa* strains that were isolated from burn wound infections.

## Material and Methods

### Bacterial strains

A total number of 57 *P*. *aeruginosa* isolates were collected from infections in burn wound patients from Shahid Motahari Hospital of Iran University of Medical Sciences, during October 2013 through March 2014. The identity of the isolates were determined with by conventional biochemical tests including Gram stain, oxidase, catalase, oxidation-fermentation (OF) test and the Kligler Iron Agar (KIA) tests [17].

### Ethics Statement

The Central Laboratory from Shahid Motahari Hospital provided the *P*. *aeruginosa* isolates for this study. The clinical information presented in this manuscript was obtained from the patient’s medical record, considering the sample type. The study protocol was approved by the Ethics Committee of Tehran University of Medical Sciences (No 25137).

### Antibiotic Susceptibility Testing

Susceptibility of isolates to various antibiotics was determined by Disk Diffusion Agar and Broth microdilution methods as recommended by the Clinical and Laboratory Standards Institute (CLSI) [18].

The following antibiotic disks (Mast Diagnostics- UK), were tested: Amikacin (AK), Gentamicin (GM), Meropenem (MEM), Imipenem (IMI), Ceftazidime (CAZ), Cefepime (CMP) and Polymixine B (PB). *Escherichia coli* ATCC 25922 was used as a control for susceptibility testing.

The MICs of Amikacin (Sigma Aldrich, St Louis, USA) and Ceftazidime (Jaber Ebne Hayyan Co, Iran) were determined by CLSI broth microdilution method (MIC range,0.5 to 256 μg/ml). *P*. *aeruginosa* ATCC 27853 were used as a control for quality assurance of the test.

### Detection of genes encoding biofilm exopolysaccharides

The genes encoding biofilm exopolysaccharides (*algD*, *pelF and pslD*) were targeted by a PCR-based method, using primers listed in Table 1 [17]. The following protocol was used for PCR procedure. DNA extraction was performed by boiling method. Each 12.5 μL reaction contains: 2.5 μL of DNA, 5 μL of Taq 2× Master Mix (Ampliqon, Denmark), 0.25 μL of each forward and reverse primers with the concentration of 10 pmol/μL and 4.5 μL of distilled water. PCR was performed under the following conditions: initial denaturation for 5 min at 95°C, then denaturation for 1 min at 95°C, 30 cycles of 40 s at 58°C (for *algD*, *pelF* and *pslB* genes), and 56°C (for *pslD* gene), 45 second at 72°C, and a final elongation step for 5 min at 72°C. PCR products were analyzed with UV light after running at 120V for 45 minute on a 1% agarose gel.

**Table 1:** Primers used for the amplification of the genes coding for biofilm exopolysaccharides among *Pseudomonas aeruginosa* isolates.

Gene	primer sequence (5^'^→3^'^)	Annealing temperature (°C)	Size of amplicon (bp)
***algD***	F-CTACATCGAGACCGTCTGCC	58	593
	R-GCATCAACGAACCGAGCATC		
***pelF***	F-GAGGTCAGCTACATCCGTCG	58	789
	R-TCATGCAATCTCCGTGGCTT		
***pslD***	F- TGTACACCGTGCTCAACGAC	56	369
	R- CTTCCGGCCCGATCTTCATC		

### Biofilm assay

*P*.*aeruginosa* isolates were inoculated in 5ml trypticase soy broth (TSB) (Gibco, USA) and incubated for 24 h at 37°C, then they were diluted in TSB to a turbidity equal to 0.5 McFarland standard and each well of a flat-bottomed polystyrene 96-well microtiter plate (Tissue culture plate 96 wells, JET BIOFIL, Canada) were inoculated with 100 μL of these dilutions. *Pseudomonas aeruginosa* ATCC 27853 and sterile broth were used as positive and negative control. After 24 h incubation at 37°C, the supernatant (containing non-adherent cells) was removed and wells were rinsed with normal saline solution (0.90% w/v of NaCl) three times. Biofilms were fixed by 96% ethanol, and then stained with crystal violet (1.5% w/v) for 20 minute, after that unbound stain was removed by washing with tap water. The dye was solubilized in 150 μL of 33% (v/v) acetic acid. The optical densities (OD) of the wells were determined by using a microplate reader (Anthos Labtec instruments, type: 22550) set to 550 nm [17]. All assays were performed in triplicate and repeated three times for each strain.

Three standard deviations above the mean absorbance of negative control were considered as cut-off OD (OD_C_). Biofilm formation was categorized by the following formulas: If OD < ODc, the biofilm was not formed (negative), If ODc < OD < 2xODc, the biofilm was weak, If 2xODc < OD < 4xODc, the biofilm was moderate. If 4xODc < OD, the biofilm was strong.

Based on sensitivity to amikacin and ceftazidime, biofilm production and genotypic characteristics, isolates were selected for further studies.

### Enzymatic assay

The biofilm detachment assay was performed as described previously [11]. Briefly, after the establishment of biofilms in the wells of a flat-bottomed polystyrene 96-well microtiter plate (Tissue culture plate 96 wells, JET BIOFIL, Canada), biofilms were incubated with different concentrations of enzymes (α-mannosidase and β-mannosidase enzyme concentrations: 0.005, 0.01, 0.015, 0.02 and 0.03 unit/ml and trypsin enzyme concentrations: 0.08, 0.175, 0.35, 0.75 and 1.5 μg/ml), the optimal concentration of each enzyme was selected and used for incubation with biofilms for 1h at 37°C. Well content was removed and washed thrice with sterile saline solution (NaCl, 0.9% w/v) and were stained using the CV assay. The optical densities (OD) of the biofilms were determined at 550 nm by using a microplate reader (Anthos Labtec instruments, type: 22550). Biofilms with no enzyme treatment was used as a positive control and medium without bacteria and enzyme was used as a negative control. The test was performed once with three replications. All enzymes were purchased from Sigma Aldrich (St Louis, USA).

Since α-mannosidase and β-mannosidase enzymes used similar buffer conditions, their combined effects on *P*. *aeruginosa* biofilm production was analyzed as well. This test was carried out once with three replications.

The bactericidal effect of the enzymes on planktonic cells of *P*. *aeruginosa* was evaluated. Firstly, 50 μL of Mueller—Hinton broth (Merck, Germany) was added to each microtiter plate well (Tissue culture plate 96 wells, JET BIOFIL, Canada) and subsequently, 50 μL of bacterial suspension with a final inoculum density of 10^8^ CFU/ml was added to each well and was mixed with α-mannosidase, β-mannosidase and Trypsin. The microtiter- plate was incubated for 20h at 37°C, and the effect of enzymes on the bacterial growth was determined with respect to well described turbidity. After evaluating the turbidity in the wells, 20 μL of the suspension with no turbidity was inoculated on the TSA media and incubated,and then presence of colonies was checked, This test was performed 3 times.

### Determination of the Minimum Biofilm Eliminating Concentrations (MBECs)

The MBECs of bacterial biofilm cultures for amikacin and ceftazidime were determined according to the method of Amorena et al using the XTT (2,3-bis[2-methyloxy-4-nitro-5-sulfophenyl]-2H-tetrazolium-5-carboxanilide) colorimetric assay with some modifications [19]. Briefly, biofilms were established in the wells of a flat-bottomed polystyrene 96-well microtiter plate (Tissue culture plate 96 wells, JET BIOFIL, Canada). After incubation of bacterial biofilms with 100 μL of serial dilutions of antibiotics at 37°C for 20 h, 50 μL of fresh XTT labeling mixture (Roche, Germany) was added to each well and subsequently incubated for 1 h at 37°C in the dark conditions [20]. The lowest concentration of the antibiotic that inhibited re-growth of the bacteria from the treated biofilm was defined as the MBEC value [21]. This test was conducted 3 times. This experiment was performed on 3 strains 1, 2 and 4, because they were susceptible to ceftazidime and amikacin in planktonic state, but strain 3 that was resistant to amikacin and strain 5 that was resistant to both amikacin and ceftazidime, were not involved in the experiment.

### The combined effect of enzymes and ceftazidime on *P*. *aeruginosa* biofilms

The combined effect of enzymes and ceftazidime on *P*. *aeruginosa* biofilms was determined as described previously [22, 23]. Briefly, bacterial biofilms with either 100 μL of ceftazidime or ceftazidime with enzyme; α-mannosidase and β-mannosidase (0.02 unit/ml) or trypsin (0.75 μg/ml) were incubated at 37°C for 20 h. Subsequently, the well content was removed and washed with normal saline. The MBEC values of ceftazidime for biofilm cultures were determined using the XTT reduction assay. This test was performed 3 times.

### Cytotoxicity assay

Cell line preparation and cytotoxicity assays were performed as described by Braydich-Stolle et al [21]. Briefly, A-431 human epidermoid carcinoma cell lines (NCBI Code: C204) were maintained in RPMI 1640 medium (Biosera, USA) supplemented with 10% heat inactivated fetal bovine serum (FBS), 2mM L-glutamine, 50 u/ml penicillin and 50 mg/ml streptomycin.

For morphological and viability studies, cells were seeded at a concentration of 5× 10^4^ cells/well in 100 μL of complete medium into 96-well plates and were incubated for 24h in a humidified atmosphere at 37°C and 6.5% CO_2_.

After 24h, when cells reached 60% confluency, selected concentrations of enzymes were added to the wells. To evaluate the cytotoxic effect of enzymes, morphological changes in cells were assessed by invert microscopy (Olympus 1x70, USA) every hour for the first 4 hours and finally after 24h.

Mitochondrial functions of the cells were evaluated by XTT reduction assay. After 24h exposing to the enzymes, specific amounts of XTT labeling mixture was directly added to the culture wells and after 4h incubation in the dark conditions, the absorbance at 492 nm was measured using a microplate reader (Anthos Labtec instruments, type: 22550).

In the present experiment, the positive control consisted of cells without enzyme exposure and for the negative control, sterile medium was used. The relative cell viability (%) was computed by this formula: [A]_test_/[A]_control_ ×100, in which [A]_test_ is the absorbance of the test sample and [A]_control_ is the absorbance of the control positive sample [24]. This test was performed 2 times in duplicates.

### Statistical analysis

Based on normal distribution of variables [i.e. ODs of biofilms before (OD B) and after (ODA) enzyme treatment and their differences (OD B-A)], non-parametric tests such as Wilcoxon Signed Ranks test and Paired-Samples T Test were used for comparison of ODs before and after treatment with enzymes and One- way ANOVA test was used for determination the effects of enzymes on different *P*.*aeruginosa* strains. A Kruskal-Wallis test was applied to study the effects of combination of mannosidase enzymes on the biofilms of strains. The differences between ceftazidime MBECs before and after using enzymes were evaluated by Mann-Whitney U test for each strain. A One- way ANOVA test was used for comparing viability (%) of the cells after enzyme assay. A *P* value <0.05 was considered statistically significant. All tests were performed using online available GraphPad software (http://www.graphpad.com).

## Results

The antimicrobial resistance of 57 *P*.*aeruginosa* isolates to amikacin (AK), gentamicin (GM), cefepime (CPM), ceftazidime (CAZ), Imipenem (IMP), meropenem (MEM) and polimyxine B (PB) were determined by disk diffusion agar method and results are listed in Table 2. The highest resistance rate was observed against CPM and GM (94.7%) and the lowest resistance rate was seen against PB (0%).

**Table 2:** Antibiotic susceptibility of the P.aeruginosa isolates by disk diffusion method.

Antimicrobial agent	Isolates, N (%)		
	Susceptible	Intermediate	Resistant
**Amikacin**	4 (7)	0	53 (93)
**Gentamicin**	3 (5.3)	0	54 (94.7)
**Cefepime**	3 (5.3)	0	54 (94.7)
**Ceftazidime**	22 (38.6)	0	35 (61.4)
**Imipenem**	5 (8.8)	3 (5.2)	49 (86)
**Meropenem**	4 (7)	0	53 (93)
**Polymixine B**	57 (100)	0	0

The frequency of genes encoding biofilm exopolysaccharides among 57 *P*.*aeruginosa* strains was as follows: *pelF* (93%), *pslD* (54.65%) and *algD* (100%). Based on the presence of these genes, 4 genotypic patterns were found, which *pelF*^*+*^, *algD*^*+*^, *pslD*^*+*^ was the most frequent pattern and 30 strains (52.63%) had this genotype and *pelF*^*-*^, *algD*^*+*^, *pslD*^*-*^ had the lowest frequency and only 2 strains (3.5%) showed this genotype (Table 3).

**Table 3:** Relative frequency of the genotypic patterns among *P*.*aeruginosa* isolates.

Genotypic pattern	Isolates, N (%)
***pelF***^***+***^**, *algD***^***+***^**, *pslD***^***+***^	30 (52.63)
***pelF***^***-***^**, *algD***^***+***^**, *pslD***^***+***^	3 (5.26)
***pelF***^***+***^**, *algD***^***+***^**, *pslD***^***-***^	22 (38.6)
***pelF***^***-***^**, *algD***^***+***^**, *pslD***^***-***^	2 (3.5)

The results of microtiter plate assay demonstrated that 55 strains (96.5%) were biofilm producers in which 30.9% of them produced strong biofilms, 47.3% produced medium biofilms and 21.8% of formed weak biofilms. Only 2 strains (3.5%) were non-producers.

The genotypic and phenotypic characteristics of 5 *P*. *aeruginosa* isolates that were selected out of 57 strains were presented in Table 4. Isolates had ability to produce moderate or strong biofilms and all of them were susceptible to polymixine B.

**Table 4:** Phenotypic and genotypic characteristics of *P*. *aeruginosa* strains were evaluated in this study.

Strain	Resistance Pattern							MIC (μg/ml)		Genotypic pattern	Biofilm
	AK	GM	CAZ	CPM	IMI	MEM	PB	AK	CAZ		
**1**	S	S	S	R	S	S	S	4	2	*pelf*^+^ *psld* ^+^ *algd*^+^	Strong
**2**	S	S	S	S	S	S	S	4	2	*pelf* ^+^ *psld* ^+^ *algd*^+^	Strong
**3**	R	R	S	R	R	R	S	256	4	*pelf*—*psld* ^+^ *algd*^+^	Moderate
**4**	S	S	S	S	S	S	S	8	2	*pelf* ^+^ *psld*—*algd*^+^	Strong
**5**	R	R	R	R	R	R	S	>256	>256	*pelf*—*psld*—*algd*^+^	Moderate

AK, amikacin; GM, gentamicin; CAZ, ceftazidime; CPM, cefepime; IMI, imipenem; MEM, meropenem; PB, polymixine B; S, sensitive; R, resistant; MIC, minimum inhibitory concentration.

**Fig 1:**
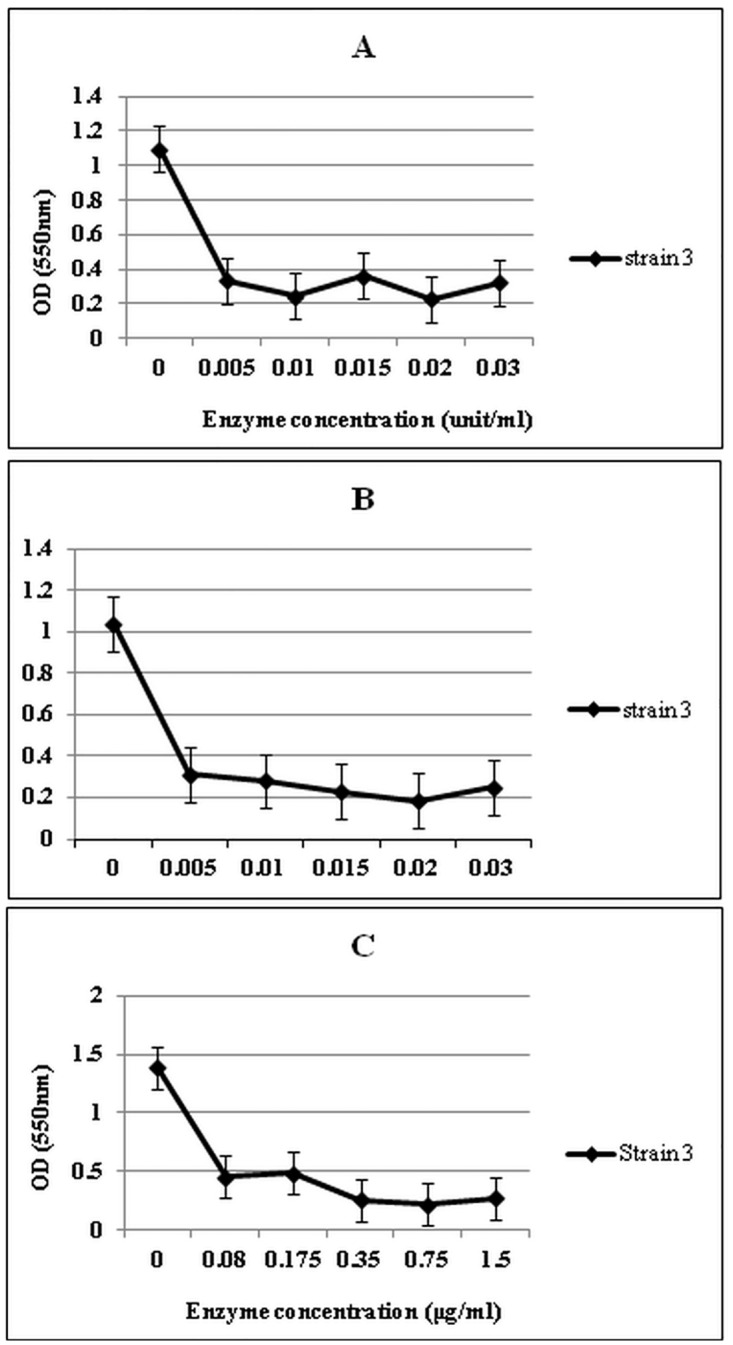
The effects of serial dilutions of alpha-mannosidase (A), beta-mannosidase (B) and trypsin (C) enzymes on the biofilm of *P*.*aeruginosa* strain 3. The experiment was done once in triplicates. Error bars represent standard errors.

**Fig 2:**
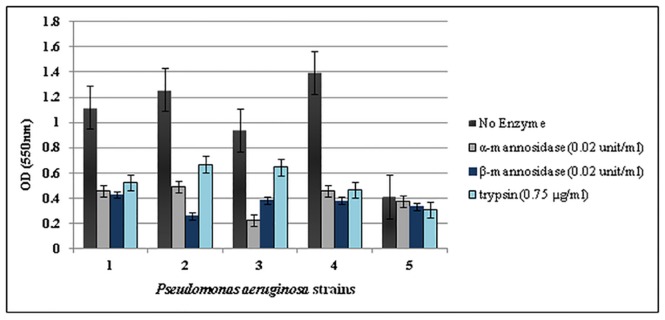
The effect of selected concentration of enzymes alpha-mannosidase, beta-mannosidase, and trypsin on the biofilms of *P*. *aeruginosa* isolates. The experiment was performed once in triplicates. Error bars represent standard errors.

**Fig 3:**
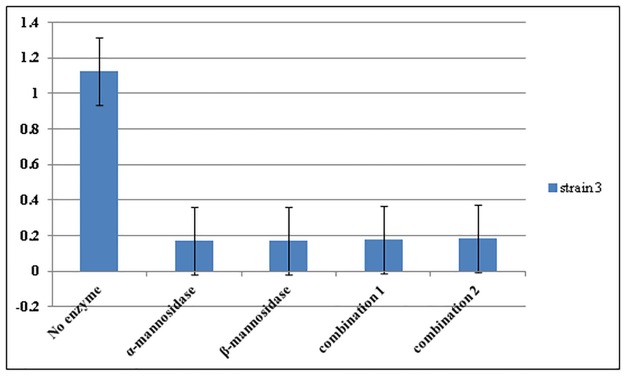
The effects of combinations ofalpha-mannosidase and beta-mannosidase enzymes on the biofilm of *P*. *aeruginosa* strain 3. alpha- mannosidase and beta- mannosidase enzymes were used at the concentration of 0.02 unit/ml. Combination1: The wells were treated with 0.02 unit/ml of enzyme alpha- mannosidase and then after 1h were treated with the same concentration of enzyme beta- mannosidase. Combination 2: The wells were treated with 0.02 unit/ml of enzyme beta- mannosidase and then after 1h were treated with the same concentration of enzyme alpha-mannosidase. The test was conducted once in triplicates. Error bars represent standard errors.

From the results of a single experiment, it was concluded that α-mannosidase, 03B2-mannosidase and trypsin enzymes reduced the ODs of the biofilms (P<0.05) (Fig 1). The most effective concentrations of α -mannosidase, β-mannosidase and trypsin enzymes on biofilms were 0.02 units/ml, 0.02 units/ml and 0.75 μg/ml, respectively (Fig 2). There were no significant differences between the effects of combinations of mannosidase enzymes with effects of each enzyme alone (P>0.05) and in both cases, the enzymes detached the biofilms (Fig 3). The β-mannosidase had no bactericidal effect, but α-mannosidase and trypsin were toxic and all tested concentrations killed bacterial cells and no turbidity was seen in the wells and no colonies were present.

The MBEC results for bacterial biofilm are listed in Table 5. All three strains were resistant to ceftazidime in biofilm; however these strains were susceptible to this agent in planktonic state (Table 4).

**Table 5:** Minimum Biofilm Eliminating Concentrations (MBECs) results for *P*. *aeruginosa* strains isolated from burn wound infections.

Strain	Amikacin (μg/ml)	Ceftazidime (μg/ml)
**1**	16	1024
**2**	16	1024
**4**	8	1024

The combination of enzymes and ceftazidime significantly decreased the ceftazidime MBECs (P<0.05) (Table 6). Biofilm did not affect the susceptibility of strains to amikacin and remained susceptible to this antibiotic.

**Table 6:** The combined effects of enzymes and ceftazidime on the MBEC values of ceftazidime.

Strain	Ceftazidime (μg/ml)	CAZ+ α-mannosidase (μg/ml)	CAZ + β-mannosidase (μg/ml)	CAZ + trypsin (μg/ml)
**1**	1024	128	128	512
**2**	1024	4	4	8
**4**	1024	4	8	32

CAZ, Ceftazidime.

**Fig 4:**
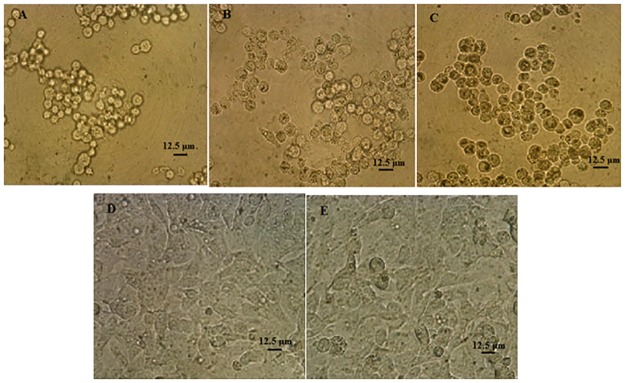
Morphology of the A-431 human epidermoid carcinoma cell lines after 24h incubation with alpha-mannosidase, beta-mannosidase and trypsin enzymes. **A.** alpha-mannosidase (0.02 unit/ml). **B.** beta-mannosidase (0.02 unit/ml). **C.** citrate buffer)100mM, pH 4.5). **D.** trypsin (0.75 μg/ml). **E.** Control positive (cells with no enzyme treatment). The experiment was performed 2 times in duplicates.

**Fig 5:**
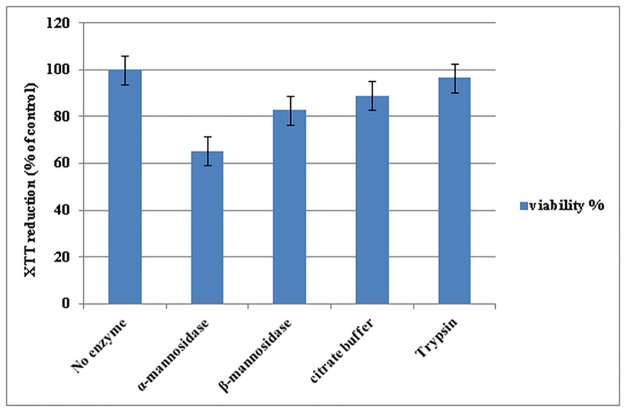
Influence of the most effective concentrations of alpha-mannosidase (0.02 unit/ml), beta-mannosidase (0.02 unit/ml), citrate buffer (100mM, pH 4.5) and trypsin (0.75μg/ml) on the viability of A-431 human epidermoid carcinoma cell lines after 24h incubation. No enzyme column represents control positive of the test. The relative cell viability (%) was computed by this formula: [A]_test_/ [A]_control_ ×100. The experiment was performed 2 times in duplicates. Error bars represent standard errors.

The morphology of the A-431 human epidermoid carcinoma cell lines after 24h incubation with trypsin enzyme had no changes compared to control cells (Fig 4) and trypsin enzyme had no significant effect on mitochondrial activity and cell viability, however the cytotoxic effect of mannosidase enzymes were high and reduced the mitochondrial function of the cells (Fig 5).

## Discussion

*P*. *aeruginosa* is one of the causes of serious infections in burn patients and emergence of multidrug resistant (MDR) isolates of *P*.*aeruginosa* in the burn units is an important problem in controlling its infections [25].

In our study, more than 90% of the isolates were resistant to amikacin, gentamicin, cefepime and meropenem and the rate of resistance to ceftazidime and Imipenem were 61% and 83%, respectively. In overall, 87% of the isolates were MDR. In a study conducted by Anvarinejad *et al*., [26], resistance level to the amikacin, gentamicin, cefepime and meropenem were similar to our study, however resistance to ceftazidime and imipenem were higher than our study (72% and 98%, respectively). Nikokar *et al*., reported much lower resistance rate for imipenem, gentamicin and amikacin and 42.3% of their isolates were MDR, which was lower than our study [27]. Probably, discrepancies in the antimicrobial resistance levels in various studies are related to the differences in the patterns of antibiotic consumption in different areas. Therefore, appropriate therapeutic regimen for treatment of *P*.*aeruginosa* infections should be selected based on the location of bacterial isolation.

According to the results, 98.4% of the isolates formed biofilm that was similar to the results of Vasiljević *et al*., [28] and Jabalameli *et al*., [17], Which reflects the importance of biofilm formation by *P*.*aeruginosa* in burn wounds and it can be considered as one of the causes of delaying the treatment of burn patients.

The prevalence of *algD* in our study was 100% and was high compared with the results of Zaranza *et al*., and Ghadaksaz *et al*., which have reported a prevalence rate of 39% and 87.5%, respectively [29, 30]. It is possible that the differences observed in the prevalence of this gene are because of different prevalent clones in each region. There is no prevalence rate about *pslD* and *pelF* genes in other regions, but studies demonstrate that *pel* gene cluster are conserved among isolates of *P*.*aeruginosa*, however *psl* genes are not present in all isolates [31], which is in agreement with our results.

In recent years, enzymatic debriding agents such as collagenase and Papain-urea are using in burn wound treatment because of their effects on collagen, elastin and fibrin that remove necrotic tissues and accelerate wound healing [32]. Regular debridement also eliminates some parts of the biofilm EPS and force the remaining bacteria to return to the state that they are metabolically active, so the antibiotics and antiseptic compounds would be more effective. In addition, the use of anti-biofilm compounds can help to eliminate biofilms from the wound bed or weaken the matrix and disintegrate the biofilm [5].

As is clear in Fig 2, both mannosidase enzymes were effective and degraded the biofilms of *P*. *aeruginosa* strains with various genotypic patterns and altered the state of biofilms from strong to the moderate or negative. Biofilm of strain 5 with genotypic pattern of *pelF*^*-*^, *algD*^*+*^, *pslD*^*-*^ was not affected (P>0.05). These results may suggest that mannosidase enzymes do not have any effect on the structure of alginate, since alginate does not have any mannose residues in its structure. The mannosidase enzymes also had same effects on ceftazidime MBECs and reduced significantly. The results of toxicity assay indicated that mannosidase enzymes cause changes in cell morphology and reduce the mitochondrial activity of the cells, and are cytotoxic.

According to our results (Fig 2), the trypsin also destroyed the *P*. *aeruginosa* biofilm; however its effect was weaker than mannosidase enzymes. These results may not be due to the poor effect of this enzyme but it may contribute to the fewer protein contents of *P*. *aeruginosa* biofilm rather than polysaccharides, since proteins are one of the sub components of biofilm in this bacterium [7]. Trypsin also decreased the ceftazidime MBECs significantly as a result of both enzymatic biofilm degradation and its bactericidal effect. The trypsin did not change the morphology or mitochondrial functions of the cells indicating its non-toxicity.

In conclusion, our finding (Fig 2) demonstrated that trypsin enzyme can be a good candidate for future studies in the field of antibiofilm agents, because it could destroy biofilms of *P*. *aeruginosa* burn isolates and result in decreased ceftazidime MBECs with no toxic side effects.

## Supporting Information

S1 File This file (XLS format) contains the raw data used in drawing of Fig 1. (XLSX)

S2 File This file (XLS format) contains the raw data used in drawing of Fig 2. (XLSX)

S3 File This file (XLS format) contains the raw data used in drawing of Fig 3. (XLSX)

S4 File This file (XLS format) contains the raw data used in drawing of Fig 5. (XLSX)
